# Explainable Neutrosophic Knowledge Distillation Model for Ocular Disease Classification Using Ultra-Wide Field Fundus Images

**DOI:** 10.3390/bioengineering13050565

**Published:** 2026-05-16

**Authors:** Nebras Sobahi, Muhammed Halil Akpınar, Salih Taha Alperen Özçelik, Abdulkadir Sengur

**Affiliations:** 1 Department of Electrical and Computer Engineering, Faculty of Engineering, King Abdulaziz University, P.O. Box 80204, Jeddah 21589, Saudi Arabia; 2 Department of Electronics and Automation, Vocational School of Technical Sciences, Istanbul University-Cerrahpasa, Istanbul 34500, Turkey; mhakpinar@iuc.edu.tr; 3 Department of Electrical-Electronics Engineering, Faculty of Engineering, Bingol University, Bingol 12000, Turkey; sozcelik@bingol.edu.tr; 4 Electrical and Electronics Engineering Department, Technology Faculty, Firat University, Elazığ 23100, Turkey; ksengur@firat.edu.tr

**Keywords:** UWF fundus imaging, retinal disease classification, NKD, deep learning, Grad-CAM++, explainable artificial intelligence

## Abstract

Ultra-wide field (UWF) fundus image classification is an important part of the entire process of medical screening and decision support. However, the discrimination of various retinal disease classes is difficult due to the similarity between classes, class imbalance, and the indeterminacy of visual patterns. In our research, an explainable neutrosophic knowledge distillation (NKD) model for UWF fundus image classification is proposed. In the proposed model, the teacher model is a ResNet50 architecture that provides the student model with supervisory information that is aware of the indeterminacy of predictions. The proposed model combines the CLAHE-based preprocessing method with the neutrosophic distillation method to enable the student model to learn from the hard labels as well as the teacher model. The experimental results were evaluated using the 5-fold cross-validation method with an additional hold-out evaluation. The experimental results show that the proposed NKD model has a mean accuracy of 84.00%, specificity of 97.33%, precision of 84.99%, recall of 84.00%, and F1-score of 84.02%. The proposed model also has an accuracy of 87.86% with specificity of 97.48% and AUC of 97.48% in the ablation-based full model evaluation. It outperformed classical machine learning baselines based on Local Binary Patterns (LBP), Histogram of Oriented Gradients (HOG), and LBP + HOG features with Support Vector Machines (SVM) classifiers, as well as the baseline student, fuzzy student, and teacher Convolutional Neural Network (CNN) models. For improved interpretability, the Grad-CAM++ technique was used to analyze the proposed NKD model. This analysis showed that the network attended to relevant retinal regions during classification. These results suggest that the proposed model can be an effective tool for UWF fundus image classification.

## 1. Introduction

Fundus imaging is a significant diagnostic tool in the monitoring of various ocular conditions [1,2]. Fundus imaging is the only modality through which blood vessels in the human body can be observed [3]. It is a diagnostic tool that can help in the early detection of various diseases such as diabetic retinopathy (DR), macular degeneration (MD), and retinal detachment. Early diagnosis of such conditions through fundus imaging can prevent a significant proportion of vision loss. However, the field of view of the traditional fundus cameras is limited. It ranges from 20 to 75 degrees. In a single scanning procedure, the cameras can image only 15% to 30% of the retinal surface. Although the cameras can produce high-quality images of the central structures of the eye such as the optic disk and the macula, the peripheral areas of the retina remain beyond the imaging capabilities of the cameras.

Therefore, it is clear that UWF image technology is used to address the physical limitations mentioned [4]. With the aid of UWF technology, it is possible to view up to 200 degrees of the retina in just one image [5]. With such an expansive view, it is possible to view nearly 82% of the surface area of the retina at once. One of the most important advantages of using UWF cameras is that it is easier to spot abnormalities in the peripheral region of the retina. For instance, it is easier to spot findings such as neovascularization and capillary occlusions. This is because these findings might not be easily visible in the normal fundus image. It is also important to note that the use of UWF fundus image technology is not only non-invasive but is also comfortable for patients. For instance, it is possible to obtain high-quality images without the need to dilate the eyes. This not only makes the process more comfortable but also ensures that it is faster. It is also important to note that the fact that UWF technology is capable of providing a lot of information makes it useful in telemedicine [6].

Although the role of UWF fundus imaging in the clinical diagnosis of diseases is significant, as the imaging technique can cover a large area of the retina in a single image, the evaluation of UWF fundus imaging is challenging. Since the UWF image contains rich, complex, and high-volume information, the evaluation of the image may require a significant time burden for specialist physicians. Moreover, visual effects may add complexity to the evaluation of the image. In order to overcome the challenges associated with the evaluation of UWF fundus imaging, the automation of clinical processes, and the reduction in subjective diagnostic errors, the integration of Deep Learning Systems (DLS) with UWF imaging has gained significant attention in recent years. By employing various preprocessing techniques, the noise in the UWF image can be reduced using artificial intelligence models, and the detection of lesions in the image can be performed rapidly and accurately, particularly in the early stages of the disease.

For instance, Liu et al. [7] proposed a classification model for grading DR in UWF images using DL techniques that incorporated hybrid preprocessing methods such as noise reduction and enhancement. Among the models trained on publicly accessible databases such as the DeepDRiD and the Kaggle databases, the Swin-S model was observed to perform the best, with an accuracy of 72%. Along similar lines, Zhang et al. [8] proposed an automated screening model for various retinal diseases such as retinal tears and DR. The model was named DeepUWF and incorporated six different preprocessing methods with weighted stacking for disease detection. The model was trained on a UWF dataset of 2644 images from three hospitals. It was observed that the model had a diagnostic accuracy of 97.45% for the detection of various diseases. In addition to the model-specific studies, review articles have highlighted the utility of UWF imaging. For instance, in their review article on the role of UWF imaging in the diagnosis and management of DR, Soliman et al. [9] highlighted the utility of the technology for the detection of various diseases in the periphery that could be missed with standard fundus photography. Based on the results from previous studies on the utility of UWF imaging for the diagnosis of DR, the review article highlighted the utility of UWF imaging with a sensitivity of 94% and specificity of 100% when compared to standard examinations. In addition to the model-specific studies, various studies have concurrently investigated the comparison between different deep learning architectures for disease classification. Nguyen et al. [10] evaluated transfer learning-based models, including ResNet152 and Vision Transformer, for distinguishing normal and diseased eyes using UWF images. In experiments conducted on an in-house dataset of 4697 images, ResNet152 showed the best performance, achieving 89.17% accuracy and an AUC of 96.47%. In a similar direction, Duan et al. [11] proposed a hybrid framework that combined a DenseNet121 feature extractor with an XGBoost classifier to diagnose 16 different conditions from UWF images while also addressing data imbalance. Trained on 10,612 images from two hospitals, the system achieved over 98.0% accuracy for common diseases and more than 99.8% accuracy for rare diseases, outperforming expert physicians.

More recently, some researchers have explored alternative classification strategies and region-focused analysis in UWF imaging. Zhang et al. [12] developed the DeepUWF+ system and compared single-stage and two-stage classification strategies for diagnosing four retinal diseases associated with abnormal fundus findings and blindness. Tested on a total of 10,541 UWF images, the two-stage strategy reduced the model error rate and achieved an average accuracy of 96.13%. Likewise, Oh et al. [13] introduced a system that focused on the traditional seven standard fields (7SF) by aligning the optic disk and macula with a U-Net model and then performing classification with ResNet-34. This approach was designed to reduce the effect of artifacts, such as eyelashes, in UWF image analysis. When evaluated on a dataset of 13,271 images, the system achieved an accuracy of 83.38%. Self-supervised learning has recently gained attention in medical image analysis because it enables deep neural networks to learn meaningful feature representations with reduced dependence on large-scale manual annotations. Instead of relying only on expert-provided labels, self-supervised methods generate supervisory signals from the data itself through contrastive learning, reconstruction, transformation prediction, or feature consistency objectives. This is particularly useful in ophthalmic imaging, where expert annotation is costly, time-consuming, and may vary between observers. A notable example is RETFound, a retinal foundation model proposed by Zhou et al. [14], which was pretrained on 1.6 million unlabeled retinal images using self-supervised learning and then adapted to multiple disease detection tasks with explicit labels. RETFound demonstrated the potential of self-supervised retinal representation learning for improving generalizability and label-efficient adaptation in ophthalmic disease detection. In this context, self-supervised representation learning provides a useful foundation for knowledge transfer, allowing the student model to benefit from richer feature information learned by the teacher network.

Knowledge distillation is a machine learning technique in which the knowledge learned by a large, complex, and high-capacity model (the teacher model) is transferred to a smaller, simpler, and more lightweight model (the student model) [15]. While the concept was originally intended for model compression, it has been found to be particularly effective for deploying high-performance deep learning systems on resource-constrained edge devices such as smartphones and handheld medical imaging devices [16]. In the process, the student model not only learns from hard labels but also from the soft labels that the teacher model predicts, which contain more information about the relationships between classes [17]. A number of studies have shown the effectiveness of the concept of knowledge distillation for ophthalmic image analysis. For instance, Abbasi et al. [18] proposed a knowledge distillation method for the classification of diabetic retinopathy (DR) in fundus images using a complex network and a simpler network with unlabeled data. Using the publicly available Messidor and EyePACS datasets, containing 4800 and 25,231 images, respectively, the student networks distilled from a VGG-based teacher achieved the best performance, with an average improvement of 6% in accuracy compared with baseline training. Similarly, He et al. [19] introduced a knowledge distillation-based deep learning strategy for classifying ocular diseases, including DR, AMD, and glaucoma, from color fundus photographs (CFP). Their method enabled the self-prediction of clinical features such as diagnosis, age, and sex. Among the models trained on the ODIR-5K dataset, which includes 5000 patient cases, the ResNet-based student networks distilled from a teacher model trained with diagnostic keywords outperformed the baseline models and achieved significant improvements in average performance scores. Beyond fundus photography, knowledge distillation has also been applied to other ophthalmic imaging modalities. Wang et al. [20] proposed a Transformer-based knowledge distillation network (TKD-Net), TKD-Net, for cortical cataract grading in slit-lamp images by combining region decomposition and sub-score modalities such as location, area, and density. Trained on an in-house dataset of 2150 images graded according to LOCS III, the student TKD-Net achieved the best performance, reaching an accuracy of 82.1% while addressing label ambiguity and missing modality issues. In another study, Islam et al. [21] developed a lightweight knowledge distillation-based deep learning model for DR classification in low-resource settings, supported by image enhancement preprocessing techniques. Using the publicly available APTOS and IDRiD datasets, containing 3662 and 516 images, respectively, the modified CBAM-supported Xception student model, distilled from a teacher model based on ResNet-152-V2 and Swin Transformer, achieved the best results, reaching 99.04% accuracy in multiclass classification on the APTOS dataset. Knowledge distillation has also been explored for segmentation tasks. Racioppo et al. [22] proposed a multi-conditional Transformer-based architecture for FAZ segmentation in OCTA images of eyes with different conditions, including healthy, Alzheimer’s disease, AMD, and DR. Their framework transferred knowledge from four different teacher encoders into a shared foundation model using the datasets obtained from the 102 eyes and the pre-trained color fundus photocoagulation images, the multi-condition model yielded the best results, obtaining a mean Dice score of 83.8%. Moya-Albor et al. in [23] presented a deep learning model based on the knowledge distillation technique for the diagnosis and classification of diabetic retinopathy (DR) lesions in the fundus photograph. The methodology of the study presented a new combination of the Kullback–Leibler (KL) and categorical cross-entropy (CCE) loss functions. The model, trained on the imbalanced dataset of the JSIEC1K (1000 images) and the balanced Messidor-2 (3592 images) datasets, obtained the best results for the distilled student model based on the Inception-V3 architecture, which yielded a validation accuracy of 97.30% on the in-house Messidor dataset. Dejene et al. in [24] presented a lightweight deep learning model for the classification of DR using the knowledge distillation technique and depth wise separable convolution. The study used the APTOS 2019 dataset (3662 images) and primary data obtained from the local eye clinics. The student model obtained the best results, which yielded 98.38% accuracy in the binary classification of the APTOS dataset. Recent studies have also investigated confidence-weighted and uncertainty-aware variants of knowledge distillation to improve the reliability of teacher–student transfer. Furlanello et al. [25] introduced Born-Again Networks, showing that a student model trained through distillation can outperform its teacher and that distillation may act as a form of regularization rather than only model compression. Their study also discussed confidence-weighted teacher supervision as a way to analyze the role of reliable teacher predictions [26]. More recent uncertainty-aware KD approaches have further shown that uncertain or low-confidence teacher outputs may be less reliable for student learning and that weighting or filtering teacher supervision according to confidence or uncertainty can improve the effectiveness of distillation. Similarly, curriculum-based KD methods, such as CTKD, adapt the distillation difficulty during training by dynamically controlling the temperature parameter [27]. These studies are closely related to the motivation of the proposed NKD framework, which also aims to modulate teacher supervision according to prediction reliability. However, unlike standard confidence-weighted KD methods that mainly depend on the maximum teacher probability, the proposed NKD formulation explicitly models teacher ambiguity through the neutrosophic indeterminacy component.

In the context of the present work, the teacher–student model for the classification of UWF fundus images will be discussed, which combines CNN with the NKD method. To be specific, the ResNet50 network will be considered the teacher network, and the ResNet18 network will be considered the student network, which will allow the developed model to balance high representational capacity with computational efficiency. Prior to training, the developed model will utilize the contrast-limited adaptive histogram equalization method to enhance the quality of the fundus images, which will allow the features of the retinal structures to be more prominent. Following the enhancement of the quality of the images, the images will be resized to 224 × 224, normalized with ImageNet statistics, and then augmented with the addition of random horizontal flips, rotations, and color jittering. Finally, the developed model will utilize the NKD method, which will allow the teacher network’s soft probabilities to be transferred to the student network. To be specific, the neutrosophic theory will be considered, which will be derived based on the indeterminacy of the teacher network’s predictions, which will be considered the difference between the two top probabilities of the teacher network or the entropy value. Thus, it is observed that the student model benefits from better guidance offered by confident teacher predictions and is less affected by uncertain predictions. Hence, it is more dependable. The effectiveness of the proposed framework is measured using metrics such as accuracy, sensitivity, specificity, precision, recall, F1-score, area under ROC curve (AUC), log loss, and expected calibration error (ECE). In addition, visualization of decisions made by the student model is achieved through Grad-CAM++ at the third residual stage.

The contributions of this paper are two-fold:

1. First, we propose a neutrosophic knowledge distillation method for UWF fundus image classification that takes into account the indeterminacy information provided by teacher models in the distillation loss function, thus making teacher–student transfer learning uncertainty-aware.
2. Second, we develop a classification framework that is efficient and follows a teacher–student paradigm, in which knowledge from a stronger teacher model is transferred to a lightweight student model to show that our uncertainty-aware distillation method is useful in fundus image classification.

## 2. Materials and Methods

In this paper, we propose an NKD framework for fundus image classification that combines a stronger teacher model, ResNet-50, with a lighter student model, ResNet-18, in order to obtain an efficient architecture without losing the benefit of rich supervisory information [28].

**Figure 1:**
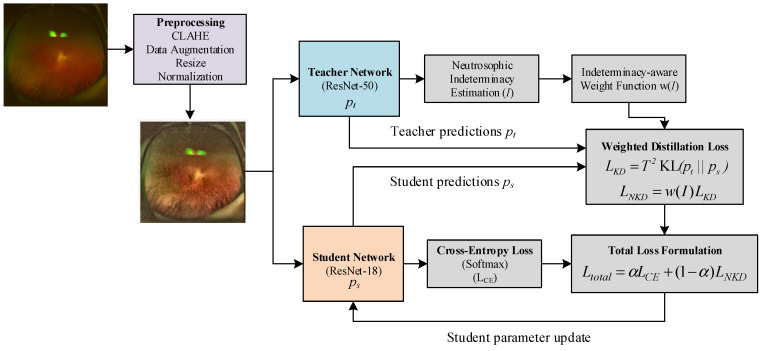
Overview of the proposed NKD framework.

Figure 1 shows the overall workflow of the proposed approach. As a first step, the input fundus image is passed through a preprocessing stage that includes CLAHE, data augmentation, resizing, and normalization, so that local retinal structures become more visible and the input distribution is better suited for network training [29]. The preprocessed image is then provided to both the teacher and student branches. On the teacher side, ResNet-50 produces soft predictions that carry not only the final class tendency but also the relative confidence across classes. These soft outputs are further used to estimate neutrosophic indeterminacy, which reflects the ambiguity of the teacher prediction. The estimated indeterminacy is then converted into an indeterminacy-aware weight, allowing the distillation process to be adjusted according to how reliable the teacher guidance is for a given sample. On the student side, ResNet-18 produces its own predictions from the same input image. These predictions are involved in two complementary objectives: the standard cross-entropy loss, which enforces consistency with the ground-truth labels, and the weighted distillation loss, which encourages the student to follow the teacher’s soft output distribution under the control of the neutrosophic weight. Finally, these two terms are combined into a unified loss function, and only the student network is updated through backpropagation. In this way, the proposed framework allows the student model to benefit from both direct label supervision and uncertainty-aware knowledge transferred from the teacher, leading to a more informative and reliable learning process.

In this framework, the teacher-guided representation transfer process allows the student network to learn not only from hard class labels but also from the soft output distribution and uncertainty-aware supervisory signals produced by the teacher model. This allows the student model to acquire richer latent feature representations beyond direct label supervision.

### 2.1. Knowledge Distillation

Knowledge distillation (KD) refers to the teacher–student learning paradigm, in which the student model is trained to mimic the performance of the teacher model, which is generally more powerful [30]. We let $z_{t}$ and $z_{s}$ denote the logit outputs of the teacher and student models, respectively. The softened class probabilities generated by the teacher model are obtained by applying the softmax function with temperature *T*:

(1) $$p_{t}^{\left(i\right)}=\frac{exp\left(z_{t}^{\left(i\right)}/T\right)}{\sum_{j=1}^{C}exp\left(z_{t}^{\left(j\right)}/T\right)},$$

where C is the number of classes. In the same way, the student distribution can be written as:

(2) $$p_{s}^{\left(i\right)}=\frac{exp\left(z_{s}^{\left(i\right)}/T\right)}{\sum_{j=1}^{C}exp\left(z_{s}^{\left(j\right)}/T\right)}.$$

The use of temperature scaling results in the softened output distribution, allowing the assimilation of not only the definite class assignment but also the similarity relationships between classes, as indicated by the teacher. Under the conventional KD framework, this transfer between the teacher and the student can be formulated in terms of the Kullback–Leibler divergence between their output distributions:

(3) $$L_{KD}=T^{2}\sum_{i=1}^{C}p_{t}^{\left(i\right)}log\frac{p_{t}^{\left(i\right)}}{p_{s}^{\left(i\right)}}.$$

At the same time, the student is also optimized with the conventional supervised classification objective. Given the ground-truth label y, the cross-entropy loss is defined as:

(4) $$L_{CE}=-\sum_{i=1}^{C}y^{\left(i\right)}log{\hat{p}}_{s}^{\left(i\right)},$$

where ${\hat{p}}_{s}$ denotes the student prediction obtained with the standard softmax operation. Hence, the overall student objective function can be written as a weighted sum of the hard label supervision term and soft label supervision term.

(5) $$L=\alpha L_{CE}+\left(1-\alpha \right)L_{KD},$$

where $\alpha \in \left[0,1\right]$ controls the relative contribution of the two losses.

### 2.2. Neutrosophic Indeterminacy

In classical neutrosophic theory, each sample can be represented by three components: truth T, indeterminacy I, and falsity F [31,32]. In the context of teacher–student knowledge distillation, these components can be derived from the softened output distribution of the teacher model. Let $p_{t}^{T}$ denote the teacher probability for class t, and let $p_{\left(1\right)}^{T}$ and $p_{\left(2\right)}^{T}$ denote the largest and second largest teacher probabilities, respectively. The truth component is defined as the degree of support provided by the teacher for the dominant class:

(6) $$T=p_{\left(1\right)}^{T}$$

The indeterminacy component measures the ambiguity of the teacher prediction. In the margin-based formulation, this ambiguity is computed from the difference between the two most probable classes:

(7) $$I=1-\left(p_{\left(1\right)}^{T}-p_{\left(2\right)}^{T}\right).$$

A small margin indicates that the teacher assigns similar probabilities to competing classes, resulting in higher indeterminacy. Alternatively, entropy-based indeterminacy can be defined as:

(8) $$I=-\frac{1}{logC}\sum_{i=1}^{C}p_{c}^{T}log\;(p_{c}^{T})$$

where C is the number of classes. The falsity component represents the degree of non-support for the dominant teacher prediction and is defined as:

(9) F=1−T

Thus, the teacher output is represented as a neutrosophic triplet (T,I,F), where T captures confidence, I captures ambiguity, and F captures complementary non-supporting evidence. Unlike simple confidence-based weighting, which only considers T, the proposed formulation explicitly incorporates the ambiguity term I when determining the contribution of each sample to the distillation loss.

### 2.3. Neutrosophic Knowledge Distillation

The proposed NKD formulation uses the indeterminacy component to control the reliability of the teacher supervision [33]. A teacher prediction with high truth and low indeterminacy should provide stronger guidance, whereas a prediction with high ambiguity should contribute less to the distillation process. Therefore, the margin-based confidence can be written as:

(10) M=1−I

and the sample-wise neutrosophic distillation weight is defined as:

(11) $$w_{i}=\sigma \left(k\left(M_{i}-\tau \right)\right),$$

where $\sigma \left(\cdot \right)$ is the sigmoid function, τ is the confidence margin threshold, and k controls the steepness of the transition. The weighted distillation loss is then formulated as:

(12) $$L_{NKD}=\frac{1}{N}\sum_{i=1}^{N}w_{i}KL(p_{T}^{i}||p_{T}^{i}).$$

Finally, the complete student objective is:

(13) $$L=\alpha L_{CE}+\left(1-\alpha \right)L_{NKD}.$$

where $L_{CE}$ is the ground-truth label loss. This formulation differs from standard confidence-weighted KD in that the weighting is not based only on the maximum teacher probability. Instead, it is derived from the neutrosophic interpretation of the teacher distribution, where ambiguity between competing classes is explicitly modeled through the indeterminacy component.

### 2.4. Dataset

The experiments conducted in this paper utilized publicly available UWF image data presented in He et al.’s work [34]. This dataset was specifically designed to more closely mimic actual-world practice by combining disease labels with image quality assessments and vital demographic data. It contains 700 high-resolution UWF images with a resolution of 3900 × 3072 pixels and was obtained with an Optos 200Tx imaging system, Optos plc, Dunfermline, Scotland at Zhejiang Provincial People’s Hospital between January 2023 and January 2024.

**Figure 2:**
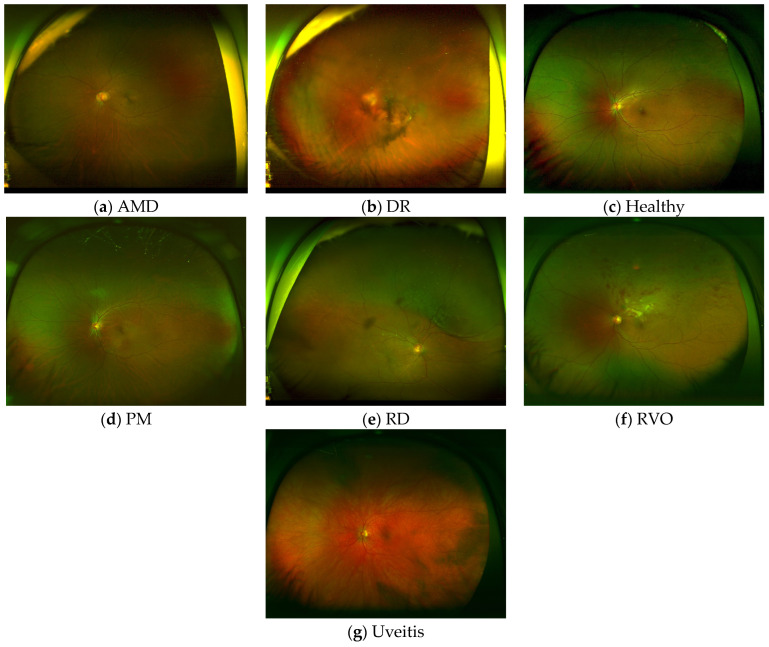
Sample UWF images for each disease class. (**a**) AMD, (**b**) DR, (**c**) Healthy, (**d**) PM, (**e**) RD, (**f**) RVO, and (**g**) Uveitis.

It is evenly split across seven classes: Diabetic Retinopathy (DR), Age-Related Macular Degeneration (AMD), Retinal Vein Occlusion (RVO), Pathological Myopia (PM), Uveitis, Retinal Detachment (RD), and healthy controls. There are 100 images in each class. Overall, it contains 689 eyes of 602 patients, with 307 female and 295 male patients. In addition to disease labels, it contains relevant metadata such as age, sex, and laterality, in addition to image quality labels such as field of view, illumination, contrast, and overall image quality. These were independently performed by three ophthalmologists and then finalized by expert review. This makes it more valuable for developing and testing robust fundus image classification techniques under realistic image quality conditions. Figure 2 displays sample UWF images for each class in the dataset.

## 3. Experimental Works and Results

The proposed method was implemented in Python using the PyCharm Community Edition 2023.3.3 development environment and run on a GPU-based system. In this study, two evaluation strategies were used for different purposes. The 5-fold stratified cross-validation was considered the primary evaluation protocol because it evaluates all samples across different folds and provides a more reliable performance estimate for the limited-size dataset. The 80/20 hold-out split was used as a supplementary evaluation to provide an additional fixed train–test assessment and to support the ablation experiments under a consistent split. Therefore, the cross-validation results are considered the main performance evidence, while the hold-out results are reported as complementary findings. Training was performed for 40 epochs with a batch size of 16 using the AdamW optimizer. The learning rate was set to 3 × 10^−4^ and the weight decay was set to 1 × 10^−4^. Mixed precision training and gradient accumulation were also used to make training more efficient and stable. In addition, a “ReduceLROnPlateau” scheduler was applied to adjust the learning rate during training. The performance of the models was evaluated using standard classification metrics, including Accuracy, Sensitivity, Specificity, Precision, Recall, and F1-score. These metrics were calculated for each fold, and the final results were reported as the mean and standard deviation across all five folds.

Table 1 shows the hyperparameter settings used to ensure the reproducibility of the proposed NKD framework. The KD temperature was set to 4.0, while the sigmoid steepness and confidence margin threshold were set to 8.0 and 0.15, respectively. The KD weight was constrained within the range of [0.10, 1.00] to avoid extremely small or large distillation contributions. In addition, the alpha value was scheduled linearly from 0.90 to 0.70 across epochs, allowing the model to initially emphasize hard-label supervision and gradually increase the contribution of the distillation loss. The CLAHE preprocessing parameters were also reported, with a clip limit of 2.0 and a tile grid size of 8 × 8.

**Figure 3:**
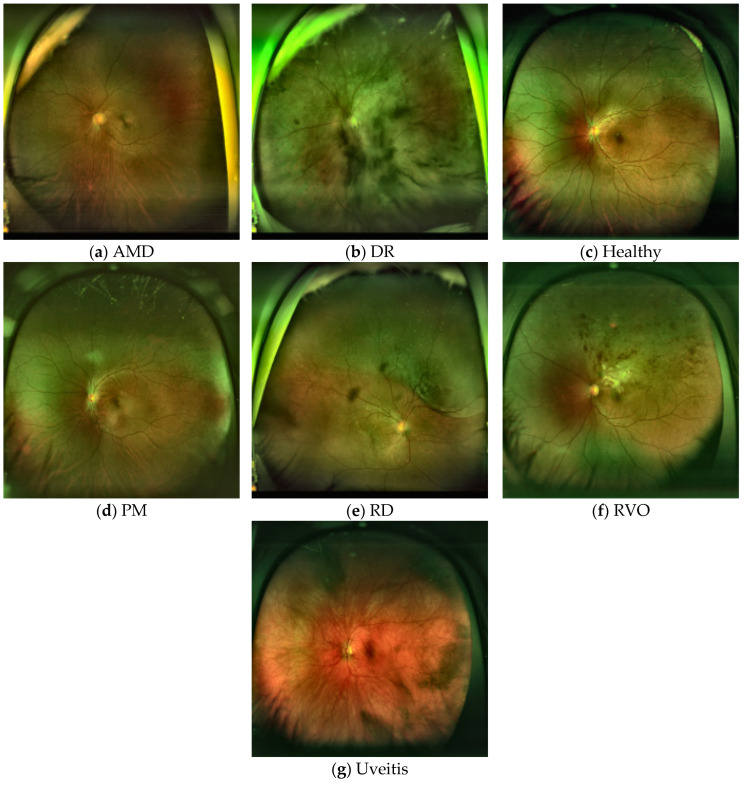
Sample UWF images for each disease class after preprocessing. (**a**) AMD, (**b**) DR, (**c**) Healthy, (**d**) PM, (**e**) RD, (**f**) RVO, and (**g**) Uveitis.

Figure 3 shows sample UWF fundus images obtained after the preprocessing stage, where CLAHE, normalization, and resizing to 224 × 224 pixels were applied. As can be seen, this preprocessing step improves local contrast and makes important retinal structures more visible, while also providing a standardized input format for the deep learning models.

**Figure 4:**
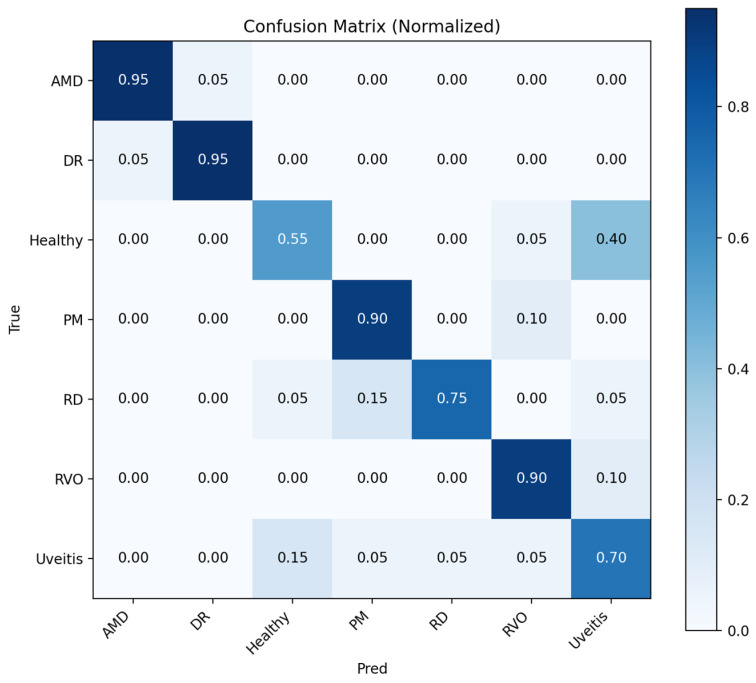
Normalized confusion matrix for teacher model.

The initial results were given for the hold-out cross-validation test where the confusion matrixes were given for teacher model and student NKD model, respectively. Figure 4 shows that the teacher model performs well for AMD, DR, PM, and RVO, whereas Healthy, RD, and Uveitis are more frequently confused. The most notable confusion occurs between Healthy and Uveitis, suggesting that visually similar retinal patterns make these categories more difficult to distinguish.

**Figure 5:**
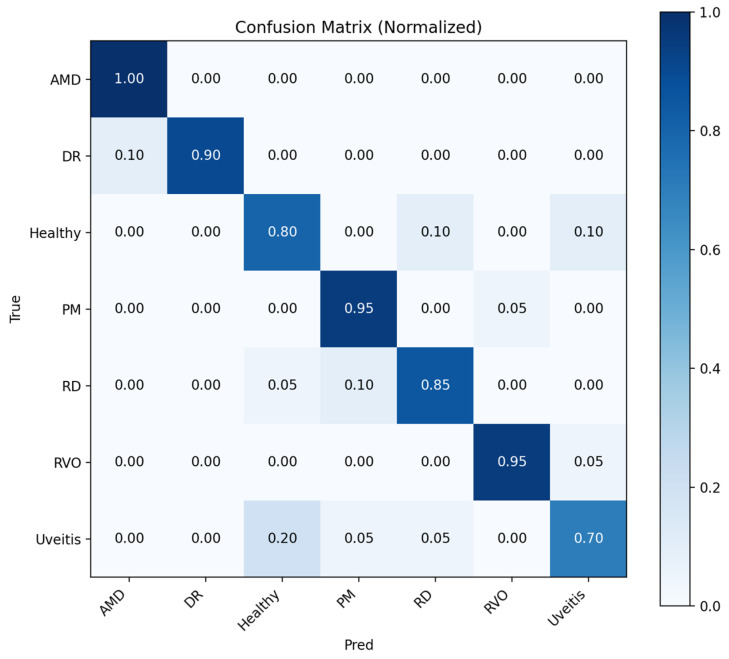
Normalized confusion matrix for student NKD model.

Figure 5 shows that the Student NKD model provides improved classification performance for most disease classes compared with the Teacher model. AMD, DR, PM, and RVO are classified with high accuracy, while Healthy, RD, and Uveitis remain relatively more challenging. The main confusion is observed between Healthy and Uveitis, indicating the visual similarity between these categories.

Table 2 shows the evaluation metrics of the achievements of both Teacher and the Student NKD models, respectively. From Table 2, we can observe that the performance of the Student NKD model outperforms that of the Teacher model in all key evaluation metrics. The accuracy improves from 0.8143 to 0.8786. The sensitivity and recall rates also show an increase from 0.8143 to 0.8786. The precision rate improves from 0.8239 to 0.8797. The F1-score also improves from 0.8141 to 0.8774. The results indicate that the performance of the student model is more balanced and reliable. The same trend can be observed in the specificity and AUC metrics. The specificity rate improves from 0.9690 to 0.9797. The results indicate that the Student NKD model has a reduced rate of false positives. The AUC rate also improves from 0.9603 to 0.9748.

**Figure 6:**
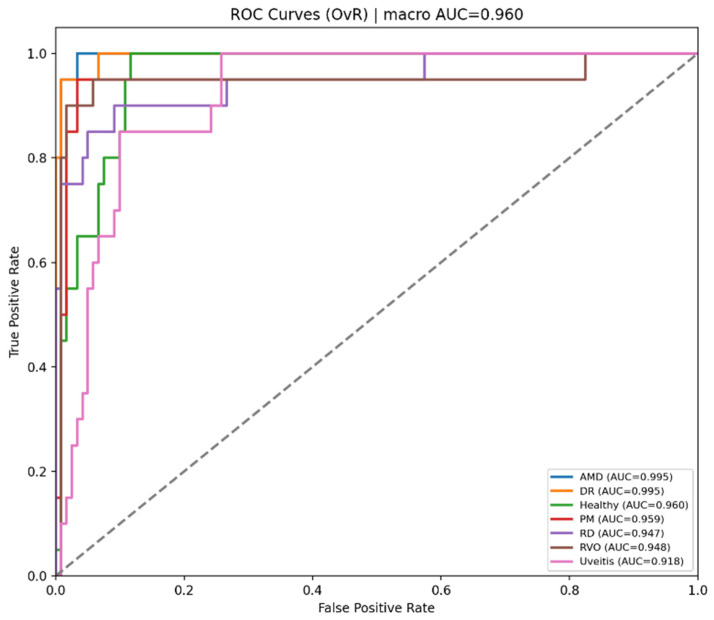
ROC curves for each class obtained with teacher model.

Figure 6 presents the one-versus-rest ROC curves of the Teacher model. The model achieves strong separability for most classes, with a macro AUC of 0.960. However, Uveitis shows the lowest class-wise AUC, suggesting that this category is more difficult to distinguish from the remaining classes.

**Figure 7:**
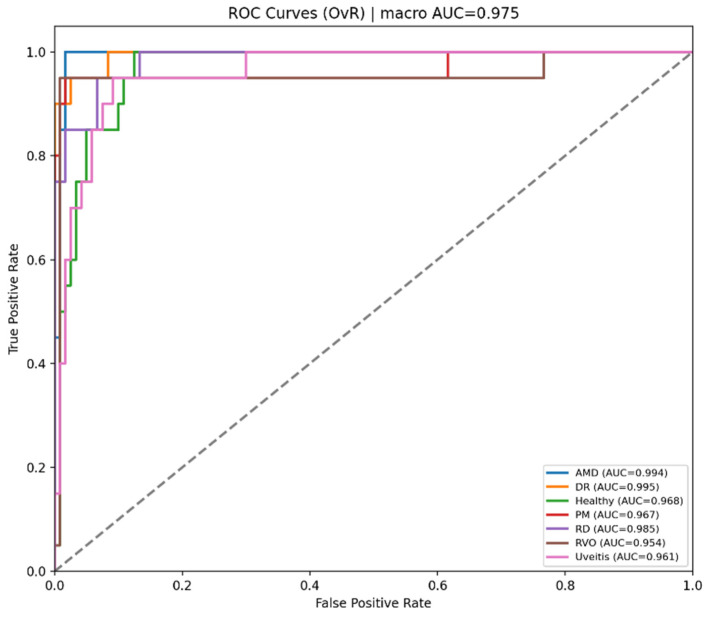
ROC curves for each class obtained with student NKD model.

Figure 7 shows the ROC curves of the Student NKD model, which achieves a higher macro AUC of 0.975 compared with the Teacher model. The curves indicate improved class-wise discrimination for most categories, although PM and RVO remain relatively lower than the other classes.

**Figure 8:**
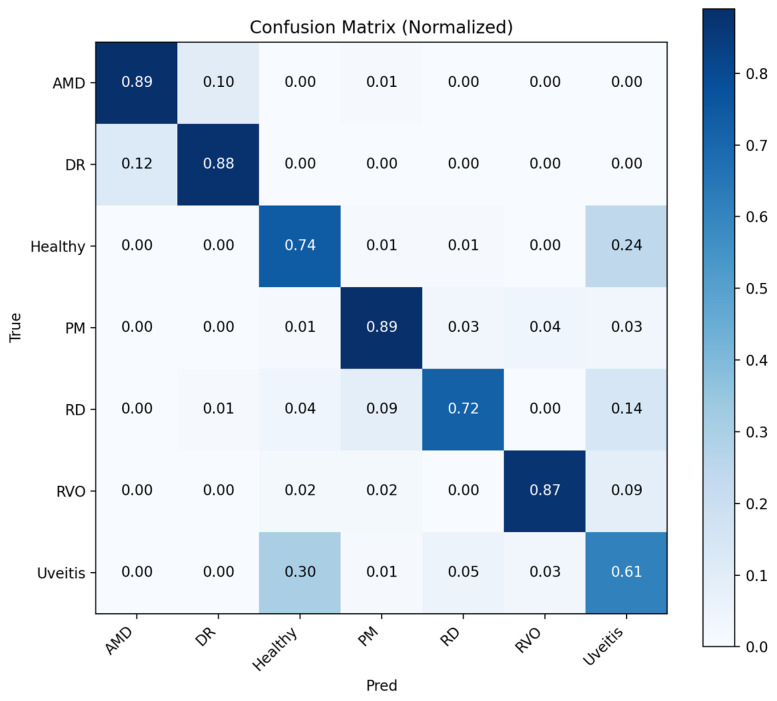
Normalized cumulative confusion matrix for teacher model.

We also conducted experiments using 5-fold cross-validation to further assess the performance of the proposed NKD method. Figure 8 shows the cumulative confusion matrix of the Teacher model under 5-fold cross-validation. AMD, DR, PM, and RVO are generally well recognized, whereas Healthy, RD, and Uveitis show more frequent misclassification. The most evident confusion occurs between Healthy and Uveitis.

**Figure 9:**
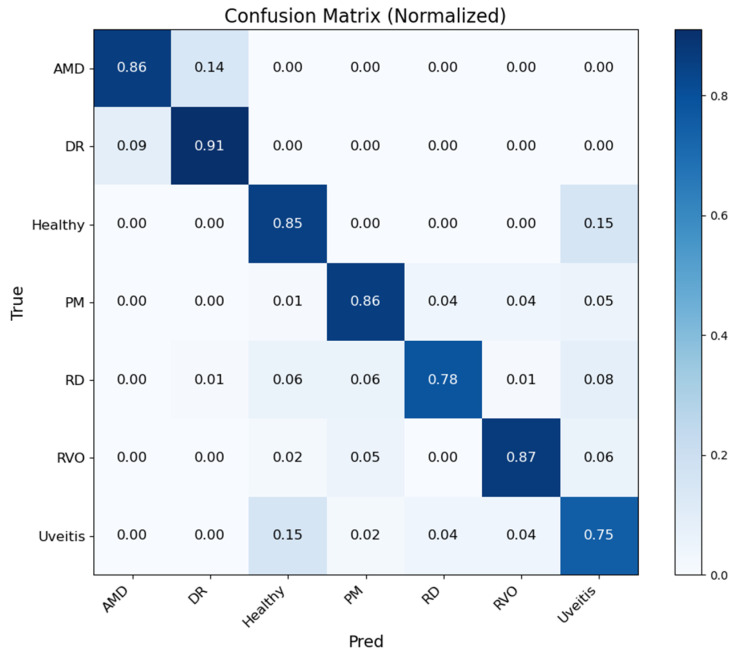
Normalized cumulative confusion matrix for student NKD model.

Figure 9 presents the cumulative confusion matrix of the Student NKD model under 5-fold cross-validation. The model maintains strong recognition for DR, AMD, PM, and RVO, while improving the classification of Healthy and RD compared with the Teacher model. Uveitis remains the most challenging class, mainly due to its overlap with Healthy and other visually similar retinal conditions.

Table 3 presents the cross-validation performance of the Teacher model over 5 folds. The accuracy of each fold ranges between 0.7429 and 0.8714, showing that performance is moderately variable between folds. The highest performance is observed in Fold 5, where accuracy is 0.8714 and F1-score is 0.8729, whereas the lowest performance is observed in Fold 4, where accuracy is 0.7429 and F1-score is 0.7434. The precision also follows a similar trend, ranging between 0.7588 and 0.8834. For the Teacher model, the 95% confidence intervals indicate a moderate variation across the five folds, particularly for accuracy, sensitivity, recall, and F1-score, while specificity remains more stable with a narrower confidence interval.

Table 4 reports the 5-fold cross-validation results for the proposed Student NKD model. The fold-wise accuracy values range from 0.8000 to 0.8857, with the strongest performance observed in Fold 2. The remaining folds stay within a relatively narrow range, indicating consistent behavior across different data splits. For the proposed Student NKD model, the 95% confidence intervals show stable performance across folds while maintaining higher mean values than the Teacher model for all evaluation metrics.

**Figure 10:**
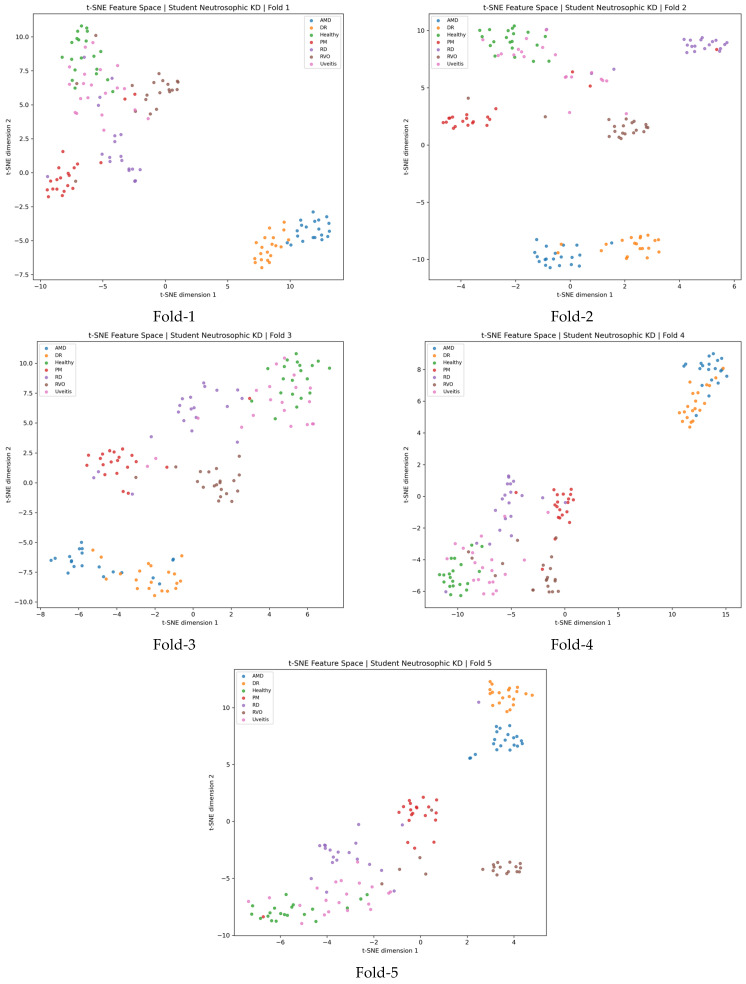
t-SNE visualization of feature representations extracted from the proposed knowledge distillation framework across five cross-validation folds.

As illustrated in Figure 10, the t-SNE projections provide a visual interpretation of the feature representations learned by the proposed *KD* framework across the five cross-validation folds. The features were extracted from the latent representation space of the student model after knowledge transfer from the teacher network. In the projected two-dimensional space, samples belonging to the same class generally appear close to each other, forming relatively compact class-specific clusters. This behavior indicates that the proposed framework does not merely improve the final classification output, but also encourages the student network to learn meaningful and discriminative internal feature representations. The separation observed among most class groups suggests that the knowledge transferred from the teacher model helps the student model preserve important structural information in the feature space. In particular, the clustering tendency across different folds demonstrates that the learned representation is relatively stable under cross-validation, rather than being specific to a single train–test split.

### 3.1. Performance Comparisons with Base Student and Fuzzy Student Models

We also made use of a baseline student model and a fuzzy student model to compare their performance against the neutrosophic student model. The baseline student was trained only on the ground truth labels using the cross entropy loss function. The fuzzy student was trained using a knowledge distillation setup where the teacher output probabilities were first mapped to fuzzy output probabilities before training the student. This would allow an evaluation of whether fuzzy or neutrosophic uncertainty improves the performance of the student model against a baseline student model that was not trained using any distillation or was trained using a normal soft target setup.

The student model’s baseline was built on a ResNet-18 architecture that was pre-trained on weights from the ImageNet dataset. The student model was trained using a supervised learning paradigm. In this regard, the student model was trained on ground truth labels by using a cross-entropy loss function. It is worth mentioning that the student model was not provided with any kind of guidance from a teacher model in the form of soft labels. In this regard, the baseline student model served as a reference model for a lightweight classifier. In addition, the student model provided a benchmark for evaluating the effectiveness of the knowledge distillation component. It is worth mentioning that the student model was trained on ground truth labels only; thus, it encapsulated the effectiveness of the student architecture regardless of any inter-class similarity information provided by the teacher model.

For the Fuzzy Knowledge Distillation (FKD), we used the same ResNet-18 backbone architecture that was trained under a knowledge distillation setting where the model was provided guidance by a ResNet-50 teacher model. Unlike the base model, the fuzzy student model leveraged information provided by hard labels as well as the softened class probability distribution provided by the teacher model. Before the knowledge distillation process, the teacher model’s class probabilities were subjected to a fuzzy transformation where each probability value was raised to a power. This ensured the dominant values were emphasized while retaining the complete distributional relationship among all classes. The fuzzy student model was optimized using a combination of cross-entropy loss and KL divergence loss. This allowed the model to leverage the information provided by the hard labels as well as the more informative class relationship information provided by the teacher model.

**Figure 11:**
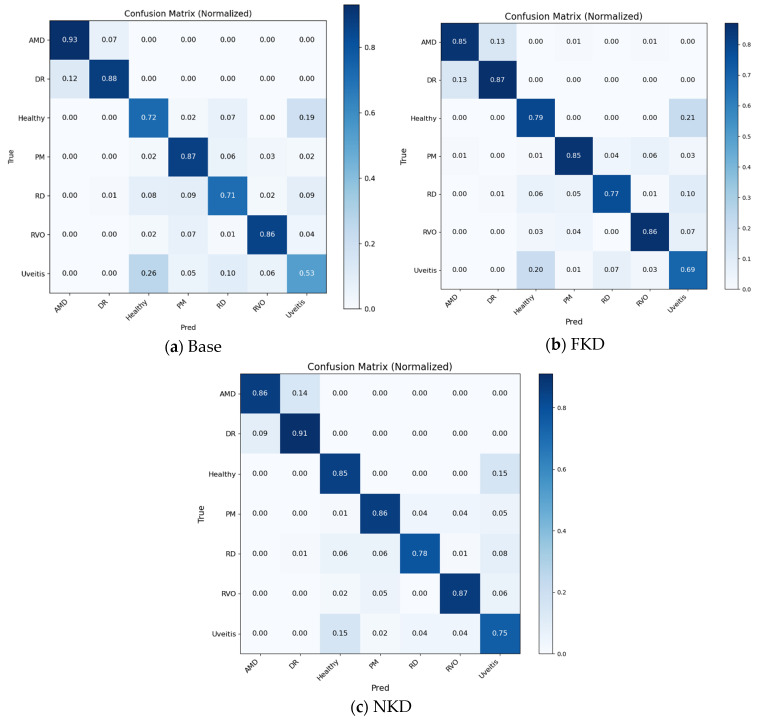
Normalized cumulative confusion matrix for student base, FKD and NKD model.

As shown in Figure 11, the comparison among the baseline student, FKD, and NKD models demonstrates that distillation improves the class-wise recognition performance of the lightweight student network. The NKD model generally provides stronger diagonal responses than the baseline and FKD models, particularly for challenging classes such as Healthy, RD, and Uveitis. The reduced confusion between Healthy and Uveitis suggests that the indeterminacy-aware weighting strategy helps the student model better handle visually similar categories. Although some classes such as AMD and PM still show minor overlap with neighboring disease categories, the cumulative confusion matrices indicate that NKD provides a more balanced classification behavior across the seven classes.

**Figure 12:**
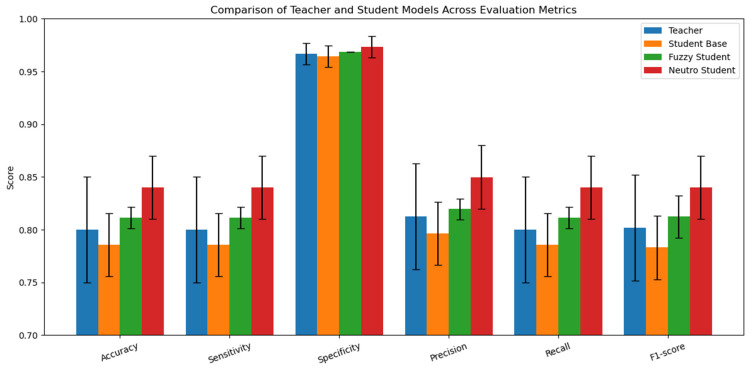
Comparison of the teacher, baseline student, fuzzy student, and neutrosophic student models in terms of mean accuracy, sensitivity, specificity, precision, recall, and F1-score over 5-fold cross-validation.

In addition, Figure 12 compares the teacher, baseline student, fuzzy student, and neutrosophic student models in terms of the mean cross-validation metrics. The NKD student achieves the highest mean values for accuracy, sensitivity, specificity, precision, recall, and F1-score, indicating that the proposed uncertainty-aware distillation strategy improves the overall performance of the lightweight student network. The error bars also show that performance varies across folds, and the statistical analysis in Table 5 further indicates that the observed improvement over the teacher should be interpreted as a performance trend rather than definitive statistical superiority.

To further assess whether the observed performance differences between the compared methods were statistically significant, paired statistical tests were conducted using the fold-wise results obtained from the 5-fold cross-validation experiments. Since all models were evaluated on the same folds, paired *t*-tests and Wilcoxon signed-rank tests were used to compare the proposed NKD model with the Teacher model using accuracy and F1-score. A significance level of *p* < 0.05 was considered statistically significant.

Table 5 presents the statistical significance analysis between the proposed Student NKD model and the Teacher model using the fold-wise results obtained from the 5-fold cross-validation experiments. The paired *t*-test and Wilcoxon signed-rank test were applied for accuracy and F1-score because the same folds were used for both models. As shown in the table, the *p*-values for accuracy were 0.1080 and 0.2500 for the paired *t*-test and Wilcoxon test, respectively. Similarly, the *p*-values for F1-score were 0.1088 and 0.3125. Since all *p*-values are greater than 0.05, the performance differences between the Student NKD model and the Teacher model are not statistically significant at the conventional significance level. Therefore, although the Student NKD model achieves higher mean accuracy and F1-score, the results should be interpreted as an improvement trend rather than definitive statistical superiority over the Teacher model.

### 3.2. Ablation Studies

In the current study, an ablation study was carried out to evaluate the impact of the individual components of the proposed neutrosophic knowledge distillation model. During the ablation study, all experiments were carried out using the same architecture for the teacher model and the student model. Additionally, the same CLAHE-based preprocessing pipeline was used. Finally, the same stratified 80/20 distribution was used to train the model. The ablation study on the proposed neutrosophic distillation mechanism included the following experiments: ce_only, neutro_full, neutro_no_weight, neutro_entropy, neutro_wmin0, and neutro_fixed_alpha. By carrying out the ablation study on the proposed model, the impact of the uncertainty estimation mode, sample-wise weighting, the minimum knowledge distillation weight, the entropy, the weight minimum set to 0, and the fixed alpha on the overall performance of the model can be evaluated.

The ablation study had the teacher model included as an upper reference baseline. The term “ce_only” was used to represent the plain ResNet-18 student network that was only trained using the cross-entropy loss. The term “neutro_full” was used to represent the full proposed methodology for training the student network. The student was learned using both hard labels and the teacher network with margin-based indeterminacy estimation and weighted neutrosophic distillation. The “neutro_no_weight” term was used to represent the full methodology minus the sample-wise weighting mechanism. The “neutro_entropy” term was used to represent the full methodology minus the margin-based indeterminacy estimation, which was replaced with entropy-based indeterminacy estimation. The “neutro_wmin0” term was used to represent the full methodology minus the minimum knowledge distillation (KD) weight. The “neutro_fixed_alpha” term was used to represent the full methodology minus the epoch-wise alpha balance. The ablation study was designed to determine which components of the proposed methodology contributed the most to the performance improvements.

Table 6 shows the ablation results for the proposed neutrosophic knowledge distillation method. Among the different versions of the method, the best results were obtained by the neutro_full version, which represents the complete version of the method. The highest performance was achieved by the complete version of the method, as indicated by the results shown in Table 6. The complete version of the method achieved 0.8786 accuracy, 0.9797 specificity, 0.8797 precision, 0.8786 recall, 0.8774 F1-score, and 0.9748 AUC, whereas the results obtained by the ce_only version were significantly lower, with 0.7929 accuracy and 0.7933 F1-score, which indicates the effectiveness of the distillation procedure. The results obtained by the variant without the sample-wise weighting mechanism were lower, with 0.8500 accuracy and 0.8489 F1-score, which indicates the effectiveness of the adaptive weighting mechanism used in the proposed method. The results were lower when the indeterminacy was calculated using the entropy-based indeterminacy, with 0.8143 accuracy and 0.8132 F1-score, whereas the results were similar when the minimum KD weight was zero. The results were better for the neutro_fixed_alpha version than the reduced versions, with 0.8357 accuracy and 0.8351 F1-score, which were lower than the results obtained by the complete version of the method.

The lower performance of the entropy-based variant may be explained by the fact that entropy measures the overall uncertainty across all classes, whereas the main ambiguity in this dataset often occurs between two visually similar classes, such as Healthy–Uveitis or AMD–DR. Therefore, entropy may be less sensitive to the dominant pairwise confusion. In contrast, the margin-based indeterminacy directly uses the gap between the two highest teacher probabilities and better captures this type of class ambiguity. This suggests that the effectiveness of the NKD framework depends on how the indeterminacy component is defined, and that the margin-based formulation is more suitable for this dataset.

### 3.3. Explainability Analysis

In order to provide interpretability support for the proposed classification framework, an explainable artificial intelligence analysis was also conducted for the proposed NKD model. To this end, visual explanations were generated for specific regions of interest in the input images that are most relevant for decision-making by the proposed model. To this end, the Grad-CAM++ method was employed for explaining the proposed NKD model. The activation maps were extracted from the last residual block of the third convolutional stage of the model architecture. This was done based on the rationale that this layer retains higher spatial information compared to other deeper layers of the model architecture, including the final layers, that are likely to contain discriminative semantic information. The XAI analysis was carried out for the proposed NKD model alone, given that the primary focus was to offer an explanation of the proposed model’s decision-making process.

To this end, a separate exemplar of Grad-CAM++ was generated for each of the test images of interest, along with visualization of ten test images per class using heatmap overlays over the original RGB fundus images. In addition to this, confidence, entropy, and indeterminacy values were also reported for each of these classes of interest, given that this would offer a broader understanding of the decision-making process of the proposed model. The primary focus of this analysis was not only to highlight specific regions of interest that are relevant for decision-making by the proposed model but also offer a broader understanding of the proposed NKD framework in relation to specific classes of interest in the context of fundus image analysis.

**Figure 13:**
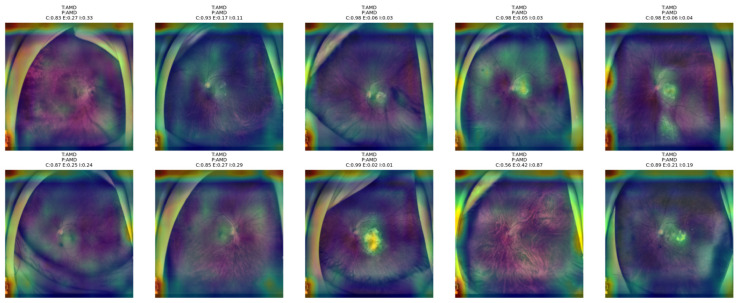
Grad-CAM++ overlay visualizations for representative AMD test images obtained from the proposed NKD model.

Figure 13 shows that the proposed NKD model primarily focuses its visual attention on the optic disk and posterior pole area in AMD cases, although some information related to a broader retinal area is also provided in some of these images. In some of these images, where confidence levels are high and entropy is low, it can be observed that the activation map is compact and focused on clinically relevant structures, indicating that the model is rendering a decisive decision based on clearer patterns of lesion development. On the other hand, in some of these images where confidence levels are low and entropy is high, it can be observed that the model’s visual attention is spread over a larger area of the retinal region, indicating that the decision process is not very clear. The image where confidence is 0.56 and indeterminacy is 0.87 is an example of this, where the heatmap is not very focused, indicating that the model is relying on a larger area of the fundus image for decision rendering. This is consistent with the uncertainty levels provided in the images above, indicating that the model’s visual attention is focused over smaller areas of the image when confidence levels are higher.

**Figure 14:**
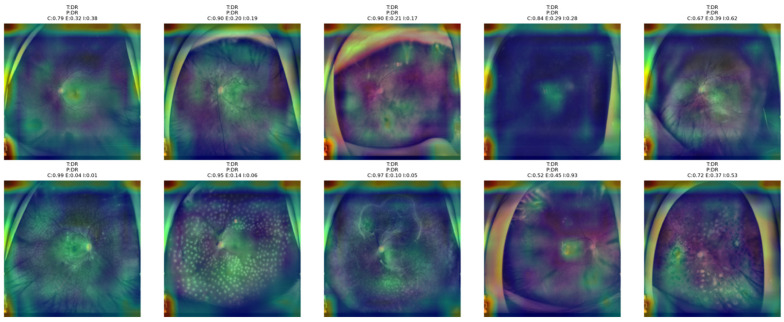
Grad-CAM++ overlay visualizations for representative DR test images obtained from the proposed NKD model.

Figure 14 shows that the proposed NKD model mainly attends to the posterior pole and lesion-related retinal regions in DR images. In samples with higher confidence, the heatmaps are more concentrated around clinically meaningful areas, suggesting a more decisive classification pattern. In contrast, in cases with lower confidence and higher entropy or indeterminacy, the attention becomes broader and more diffuse across the fundus. This behavior suggests that the model focuses more selectively when DR findings are clearer and relies on a wider retinal context when the decision is more uncertain.

**Figure 15:**
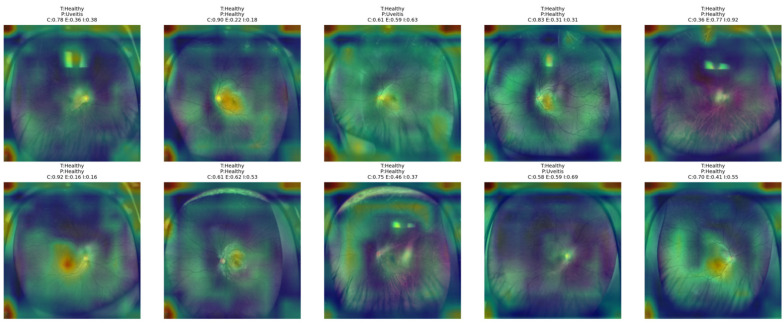
Grad-CAM++ overlay visualizations for representative Healthy test images obtained from the proposed NKD model.

Figure 15 shows that the proposed NKD model focuses its attention mainly on the optic disk and central retinal structures for healthy images. In the correctly classified images with higher confidence, the activation maps are more concentrated around the center, reflecting a consistent recognition pattern. However, for images with lower confidence, the highlighted areas are more dispersed and lack focus over the fundus image. This analysis indicates that the model primarily recognizes healthy images due to the absence of significant pathological features, while still relying on central anatomical structures for the decision-making process.

**Figure 16:**
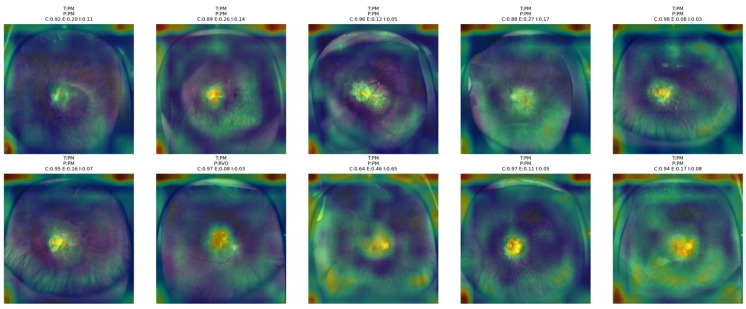
Grad-CAM++ overlay visualizations for representative PM test images obtained from the proposed NKD model.

From Figure 16 below, it is evident that the proposed NKD model mainly focuses on the macular and central retina regions of PM images, as expected based on the typical locus of pathological changes for this group. In many cases where the images are classified correctly, it is obvious that the attention maps are concentrated around the central lesion site. This suggests that the proposed model is making classifications based on local structural changes. However, for some images where the PM patterns are not so clear, the highlighted regions are extended to the general retina region.

**Figure 17:**
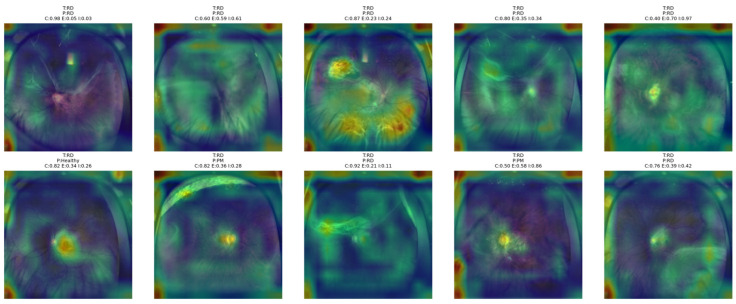
Grad-CAM++ overlay visualizations for representative RD test images obtained from the proposed NKD model.

As shown in Figure 17, the attention of the proposed NKD model is focused on the central as well as peripheral areas of the RD images rather than a particular compact area. In the case of correctly classified images, the activation maps show a wider area of abnormalities. This is logical since the area of the retina is generally affected in the case of the correct class. However, the attention maps show a disorganized attention distribution for the incorrectly classified images. This shows that the model is having difficulty in distinguishing the RD from visually related classes.

**Figure 18:**
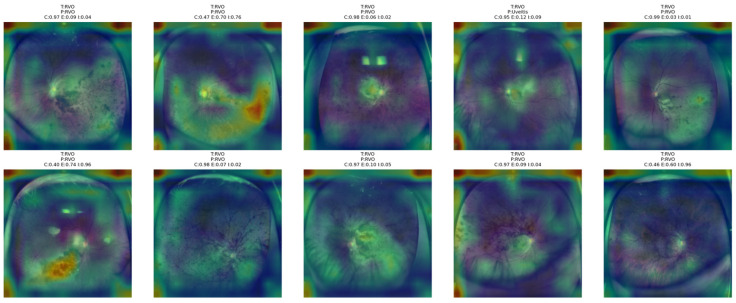
Grad-CAM++ overlay visualizations for representative RVO test images obtained from the proposed NKD model.

Figure 18 shows that the proposed NKD model tends to mainly concentrate on the optic disk, macular area, and nearby vascular abnormalities for RVO images. In correctly classified images, the attention maps are normally more concentrated around the optic disk, macular area, and nearby vascular abnormalities. This implies that the proposed NKD model is based on meaningful structures of the retina for RVO images. In images with lower confidence or higher uncertainty, the highlighted areas are normally more diffuse and can be extended to the surrounding retina.

**Figure 19:**
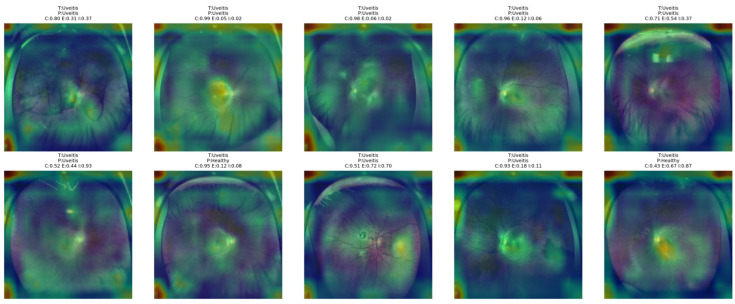
Grad-CAM++ overlay visualizations for representative Uveitis test images obtained from the proposed NKD model.

Figure 19 demonstrates that the proposed NKD model appears to be focusing on the central part of the retinal area and surrounding inflammatory areas in Uveitis images. In correctly classified images, it appears that the model is paying closer attention to these regions of interest, indicating that it is picking up on some relevant visual cues for this class of images. In less certain or misclassified images, these regions of interest appear to be broader and disorganized, covering a larger area of the retinal area of the image. This would indicate that Uveitis is one of the more difficult classes for this model, even when considering a broader area of the image.

The Grad-CAM++ analysis in this study was used as a qualitative interpretability tool to visualize the retinal regions that contributed to the model decisions. A quantitative evaluation of the explanation maps, such as pointing game accuracy or Dice overlap with lesion masks, could not be performed because the dataset provides image-level diagnostic labels but does not include pixel-level lesion annotations or lesion point coordinates. Therefore, the present Grad-CAM++ results should be interpreted as qualitative evidence of model attention rather than a quantitatively validated localization analysis. Future work should evaluate the generated explanation maps using lesion-level annotations and quantitative interpretability metrics, including pointing game accuracy, dice overlap, and insertion/deletion scores.

## 4. Discussions

In this study, we propose an explainable NKD approach for UWF fundus image classification by leveraging the supervisory signal from a ResNet50 teacher to a lightweight ResNet18 student after applying CLAHE-based preprocessing to the 224 × 224 images. The end-to-end training allows the student to learn the inter-class relationships from the hard labels as well as the soft labels provided by the teacher model without relying on any hand-crafted retinal descriptors. When evaluated on the hold-out set, the proposed NKD student model reported 87.86% accuracy, 87.86% sensitivity, 97.97% specificity, 87.97% precision, 87.86% recall, 87.74% F1-score, and 97.48% AUC values compared to the teacher model that reported 81.43% accuracy and 81.41% F1-score. When evaluated on the 5-fold cross-validation approach, the NKD model reported stability with mean accuracy, sensitivity, and recall values of 84.00%, specificity of 97.33%, precision of 84.99%, and F1-score of 84.02%. The results were more consistent for DR, AMD, PM, and RVO, while the Uveitis class remained the most challenging due to the presence of overlap with the Healthy as well as the retinal disease classes. Additional comparisons with the baseline and fuzzy student models, together with the ablation results, showed that the proposed NKD formulation produced the strongest performance, while Grad-CAM++ visualizations indicated that the model focused on clinically meaningful retinal regions during decision making.

The observation that the Student NKD model outperforms the ResNet50 teacher requires careful interpretation. This behavior may be attributed to the higher risk of overfitting of the larger teacher model on the relatively small dataset, while the lightweight ResNet18 student may provide a better capacity-to-data-size balance. In addition, knowledge distillation can act as a regularization mechanism by exposing the student to softened inter-class relationships rather than only hard labels. In the proposed NKD framework, the indeterminacy-aware weighting strategy may further reduce the influence of ambiguous teacher predictions and emphasize more reliable supervisory signals. Therefore, the superior mean performance of the student model should be interpreted as the combined effect of model capacity, regularized learning, and uncertainty-aware knowledge transfer, rather than as evidence that the student architecture is inherently stronger than the teacher.

In the comparison experiments, we also included several classical machine learning baselines in order to provide a more conventional reference against the proposed deep learning framework. In this pipeline, all UWF fundus images were first resized to 224 × 224 pixels and preprocessed with CLAHE to enhance local contrast. After preprocessing, the images were converted to grayscale and represented using handcrafted descriptors rather than learned deep features. Three feature settings were evaluated: LBP, HOG, and the concatenation of LBP and HOG features. LBP was extracted with a radius of 1 using an 8-neighbor formulation, and the resulting representation was summarized as a 256-bin normalized histogram. For HOG, the descriptor was computed with a window size of 128, block size of 16, block stride of 8, cell size of 8, and 9 orientation bins. These settings were selected to capture complementary texture and structural information from retinal images.

For classification, each feature representation was evaluated with two support vector machine variants, namely a linear-kernel SVM and an RBF-kernel SVM. In all cases, the classifier was implemented within a pipeline that first applied standardization through standard scaler and then trained an SVM with C set to 1.0, gamma set to scale, probability estimation enabled, and class weight set to balanced in order to partially compensate for class imbalance. As a result, six classical comparison methods were tested in total: LBP + linear SVM, LBP + RBF SVM, HOG + linear SVM, HOG + RBF SVM, LBP + HOG + linear SVM, and LBP + HOG + RBF SVM. Their performance was assessed under 5-fold stratified cross-validation using the same seed value of 42, and the reported metrics included accuracy, specificity, precision, recall, F1-score, AUC, and log-loss.

As shown in Table 7, the performance of the suggested NKD model is significantly better than that of all other methods. The accuracy, recall, and F1-score of the suggested model are 0.8400, 0.8400, and 0.8402, respectively. The suggested model outperforms both the classical machine learning methods and the CNN-based reference methods. The performance of the suggested NKD model in terms of specificity is also the highest at 0.9733. The precision of the suggested model is 0.8499. The results show that the suggested distillation method has a clear advantage over the classical machine learning methods and the reference CNN-based methods. The results show that the suggested method is better than the SVM method based on the handcrafted feature set and both the reference CNN-based student and teacher models. The performance of the classical machine learning methods is optimal for the LBP-SVM method using an RBF kernel. The accuracy and F1-score of the LBP-SVM method using an RBF kernel are 0.6457 and 0.6397, respectively. The performance of the classical machine learning methods using the HOG method is slightly weaker than that using the LBP method. The combination of the LBP and HOG methods slightly improves the performance of the linear SVM method. The performance of the suggested ResNet-18 model based on the student method is significantly better than that of all the classical machine learning methods. The accuracy and F1-score of the suggested model are 0.7857 and 0.7831, respectively. The performance of the suggested ResNet-50 model based on the teacher method is slightly better than that of the suggested ResNet-18 model. The accuracy and F1-score of the suggested model are 0.8000 and 0.8018, respectively. The results show that the suggested model is better than both the suggested student and teacher models. The results show that the suggested method is effective in helping the suggested student model learn better than the suggested teacher model.

The advantages of the proposed NKD framework are as follows:

1. It allows a lightweight student model to learn from the uncertainty guidance provided by a teacher model, thus improving classification results compared to traditional supervised learning methods for the student model.
2. It produces better results compared to traditional machine learning baselines and baseline CNN methods, thus proving the effectiveness of the proposed method for improving classification results in UWF fundus image classification tasks.
3. It provides better decision support results compared to traditional methods by using Grad-CAM++ visualizations that highlight clinically relevant retinal areas used by the proposed method for classification.

The disadvantages of the proposed method are as follows:

1. The proposed method is complex compared to traditional supervised learning methods since it requires the teacher model to be trained along with additional distillation components.
2. The proposed method requires several hyperparameters that affect the results, including temperature, alpha scheduling, and weights, which may need tuning for optimal results.
3. Visually similar disease classes are still difficult to distinguish from each other; therefore, inter-class confusion cannot be fully eliminated.

The present study formulates UWF fundus image analysis as a multi-class classification problem because each image in the used dataset is assigned a single primary diagnostic label. This setting is appropriate for evaluating the proposed method under the available annotation protocol. However, in real clinical scenarios, multiple retinal abnormalities may coexist in the same eye, making multi-label classification more suitable for comprehensive disease screening. Therefore, extending the proposed framework to multi-label classification is an important direction for future work.

The present study has an important limitation regarding dataset size and external generalizability. The experimental evaluation was conducted on a single publicly available UWF fundus dataset containing 700 images, with 100 images per class, collected from one institution. Although 5-fold stratified cross-validation was used to obtain a more reliable internal evaluation, the absence of an independent external validation dataset limits the strength of generalization claims. In particular, the hold-out test set contains a relatively small number of samples per class, which may increase the variability of per-class estimates. Therefore, the reported results should be interpreted within the constraints of the available dataset. External validation using larger multi-center UWF or fundus datasets is required to further confirm the robustness and clinical generalizability of the proposed framework.

Although the Grad-CAM++ heatmaps provide visual insight into the regions used by the model during classification, they were not formally validated by ophthalmologists in the current study. Therefore, the interpretation that the model focuses on clinically meaningful retinal regions should be considered qualitative and exploratory. Future studies should include systematic ophthalmologist assessment of representative heatmaps, preferably with multiple experts, to confirm the clinical relevance of the highlighted regions.

## 5. Conclusions

In this work, we extend the proposed NKD framework for improving UWF fundus image classification by incorporating uncertainty-based knowledge distillation from a ResNet50 teacher model to a lightweight ResNet18 student model. In addition, we leverage the effectiveness of CLAHE-based image preprocessing by incorporating neutrosophic distillation for a more informative knowledge distillation from the teacher model. The results from the experiment indicate that the proposed NKD model has a better performance compared to classical machine learning algorithms, the proposed student model, the proposed fuzzy student model, and even the teacher model for all the principal evaluation metrics. In addition, the ablation study indicates that the proposed neutrosophic formulation has the best performance compared to other configurations, whereby the performance decreases when the proposed formulation is modified by removing the adaptive weights, modifying the proposed uncertainty formulation, or by using a constant value for alpha balance. Moreover, the results from the proposed model indicate that the decision-making process is based on clinically relevant parts of the retinal image, thus supporting the interpretability of the proposed model for improving confidence in the decision-making process.

Future work should validate the proposed framework on larger external and multi-center UWF or fundus datasets to better assess its generalization ability across different imaging conditions and clinical populations. The framework can also be extended to multi-label classification, since multiple retinal abnormalities may coexist in real clinical scenarios. In addition, future studies should investigate multimodal learning by incorporating clinical metadata with retinal images and should evaluate more deployment-oriented lightweight student networks for clinical screening systems. Moreover, we will focus on validating the proposed framework on larger external and multi-center UWF or fundus datasets to better assess its generalization ability under different imaging conditions and clinical populations.

## Figures and Tables

**Table 1:** Hyperparameter settings used for reproducibility of the proposed NKD framework.

Parameter	Value
KD temperature, T	4.0
Sigmoid steepness, k	8.0
Confidence margin threshold, τ	0.15
KD weight lower bound, w_min	0.10
KD weight upper bound, w_max	1.00
Alpha scheduling strategy	Linear epoch-wise decay
Initial alpha, α_start	0.90
Final alpha, α_end	0.70
CLAHE clip limit	2.0
CLAHE tile grid size	8 × 8
Optimizer	AdamW
Learning rate	3 × 10^−4^
Weight decay	1 × 10^−4^
Batch size	16
Number of epochs	40

**Table 2:** Performance evaluation metrics for Teacher and Student NKD models.

Method	Accuracy	Sensitivity	Specificity	Precision	Recall	F1-Score	AUC
Teacher	0.8143	0.8143	0.9690	0.8239	0.8143	0.8141	0.9603
Student NKD	0.8786	0.8786	0.9797	0.8797	0.8786	0.8774	0.9748

**Table 3:** 5-fold cross-validation results for the Teacher model.

Fold	Accuracy	Sensitivity	Specificity	Precision	Recall	F1-Score
1	0.7571	0.7571	0.9595	0.7735	0.7571	0.7615
2	0.8143	0.8143	0.9690	0.8256	0.8143	0.8174
3	0.8143	0.8143	0.9690	0.8209	0.8143	0.8137
4	0.7429	0.7429	0.9571	0.7588	0.7429	0.7434
5	0.8714	0.8714	0.9786	0.8834	0.8714	0.8729
Mean ± Std	0.8000 ± 0.05	0.8000 ± 0.05	0.9667 ± 0.01	0.8125 ± 0.05	0.8000± 0.05	0.8018 ± 0.05
95% CI	0.7361–0.8639	0.7361–0.8639	0.9560–0.9773	0.7514–0.8735	0.7361–0.8639	0.7382–0.8653

**Table 4:** 5-fold cross-validation results for the proposed student NKD model.

Fold	Accuracy	Sensitivity	Specificity	Precision	Recall	F1-Score
1	0.8286	0.8286	0.9714	0.8442	0.8286	0.8276
2	0.8857	0.8857	0.9810	0.8883	0.8857	0.8850
3	0.8000	0.8000	0.9667	0.8151	0.8000	0.8011
4	0.8143	0.8143	0.9690	0.8248	0.8143	0.8158
5	0.8714	0.8714	0.9786	0.8771	0.8714	0.8714
Mean ± Std	0.8400 ± 0.03	0.8400 ± 0.03	0.9733 ± 0.01	0.8499 ± 0.03	0.8400 ± 0.03	0.8402 ± 0.03
95% CI	0.7941–0.8859	0.7941–0.8859	0.9657–0.9810	0.8102–0.8896	0.7941–0.8859	0.7951–0.8852

**Table 5:** Statistical significance analysis between the proposed Student NKD model and Teacher model based on 5-fold cross-validation.

Comparison	Metric	Paired *t*-Test *p*-Value	Wilcoxon *p*-Value	Interpretation
NKD vs. Teacher	Accuracy	0.1080	0.2500	Not significant
NKD vs. Teacher	F1-score	0.1088	0.3125	Not significant

**Table 6:** Ablation results of the proposed NKD framework.

Method	Accuracy	Sensitivity	Specificity	Precision	Recall	F1-Score	AUC
teacher	0.8143	0.8143	0.9690	0.8239	0.8143	0.8141	0.9603
ce_only	0.7929	0.7929	0.9655	0.7987	0.7929	0.7933	0.9607
neutro_full	0.8786	0.8786	0.9797	0.8797	0.8786	0.8774	0.9748
neutro_no_weight	0.8500	0.8500	0.9750	0.8513	0.8500	0.8489	0.9677
neutro_entropy	0.8143	0.8143	0.9690	0.8130	0.8143	0.8132	0.9583
neutro_wmin0	0.8143	0.8143	0.9690	0.8111	0.8143	0.8103	0.9690
neutro_fixed_alpha	0.8357	0.8357	0.9726	0.8441	0.8357	0.8351	0.9684

**Table 7:** Performance comparison of the proposed NKD with Machine learning methods and ResNet18 and ResNet50 CNN models for UWF fundus image classification task.

Method	Accuracy	Specificity	Precision	Recall	F1-Score
LBP SVM LINEAR	0.5957 ± 0.03	0.9326 ± 0.01	0.5983 ± 0.03	0.5957 ± 0.03	0.5922 ± 0.03
LBP SVM RBF	0.6457 ± 0.04	0.9410 ± 0.01	0.6482 ± 0.04	0.6457 ± 0.04	0.6397 ± 0.04
HOG SVM LINEAR	0.6029 ± 0.04	0.9338 ± 0.01	0.6025 ± 0.03	0.6029 ± 0.04	0.5965 ± 0.04
HOG SVM RBF	0.6100 ± 0.05	0.9350 ± 0.01	0.6076 ± 0.05	0.6100 ± 0.05	0.5983 ± 0.05
LBPHOG SVM LINEAR	0.6229 ± 0.03	0.9371 ± 0.01	0.6240 ± 0.03	0.6229 ± 0.03	0.6171 ± 0.03
LBPHOG SVM RBF	0.6229 ± 0.04	0.9371 ± 0.01	0.6175 ± 0.03	0.6229 ± 0.04	0.6122 ± 0.03
Student-base (ResNet18)	0.7857 ± 0.03	0.9643 ± 0.01	0.7964 ± 0.03	0.7857 ± 0.03	0.7831 ± 0.03
Teacher (ResNet50)	0.8000 ± 0.05	0.9667 ± 0.01	0.8125 ± 0.05	0.8000± 0.05	0.8018 ± 0.05
Student NKD	0.8400 ± 0.03	0.9733 ± 0.01	0.8499 ± 0.03	0.8400 ± 0.03	0.8402 ± 0.03

## Data Availability

Data is publicly available at https://springernature.figshare.com/articles/dataset/Open_ultrawidefield_fundus_image_dataset_with_disease_diagnosis_and_clinical_image_quality_assessment/26936446?file=49014559 (accessed on 2 April 2026).
